# Retraction: Effect of Fasudil on remyelination following cuprizone‐induced demyelination.

**DOI:** 10.1111/cns.13393

**Published:** 2020-05-27

**Authors:** Jing Wang, Ruo‐Xuan Sui, Qiang Miao, Qing Wang, Li‐Juan Song, Jie‐Zhong Yu, Yan‐Hua Li, Bao‐Guo Xiao, Cun‐Gen Ma

**Affiliations:** ^1^ Department of Neurology, First Affiliated Hospital Shanxi Medical University Taiyuan China; ^2^ The Key Research Laboratory of Benefiting Qi for Acting Blood Circulation Method to Treat Multiple Sclerosis of State Administration of Traditional Chinese Medicine Shanxi University of Traditional Chinese Medicine Taiyuan China; ^3^ Institute of Brain Science Shanxi Datong University Datong China; ^4^ Institute of Neurology, Huashan Hospital, Institutes of Brain Science and State Key Laboratory of Medical Neurobiology Fudan University Shanghai China

## Abstract

Retraction: Wang J, Sui R‐X, Miao Q, et al. Effect of Fasudil on remyelination following cuprizone‐induced demyelination. *CNS Neuroscience & Therapeutics*, 2020;26:76–89. https://doi.org/10.1111/cns.13154. The above article published online on May 23, 2019, in Wiley Online Library (wileyonlinelibrary.com), has been retracted by agreement between the authors, the journal Editor in Chief, Professor Jun Chen, and John Wiley & Sons Ltd. The retraction has been agreed due to major overlap with a previously published article from the same group of authors.

Retraction: Wang J, Sui R‐X, Miao Q, et al. Effect of Fasudil on remyelination following cuprizone‐induced demyelination. *CNS Neuroscience & Therapeutics*, 2020;26:76–89. https://doi.org/10.1111/cns.13154. The above article published online on May 23, 2019, in Wiley Online Library (wileyonlinelibrary.com), has been retracted by agreement between the authors, the journal Editor in Chief, Professor Jun Chen, and John Wiley & Sons Ltd. The retraction has been agreed due to major overlap with a previously published article from the same group of authors.

